# Neonatal mortality and associated factors in Ethiopia: a cross-sectional population-based study

**DOI:** 10.1186/s12905-021-01308-2

**Published:** 2021-04-16

**Authors:** Habtamu Dessie Mitiku

**Affiliations:** Department of Statistics, College of Natural and Computational Science, Injibara University, Injibara, Amhara Ethiopia

**Keywords:** Women's autonomy, Neonate, Mortality, Ethiopia

## Abstract

**Background:**

The neonatal period is the most critical time of human life for diseases. Neonatal morbidity and mortality are significant contributors to under-five morbidity and mortality in a low-income country like Ethiopia. Women are one of the key actors for the improvement of maternal, neonatal, and child healthcare utilization. However, there's no evidence on the association of women’s decision-making autonomy with neonate death at a national level in Ethiopia. Therefore, this study aimed to assess the neonatal mortality and associated factors in Ethiopia.

**Methods:**

A total of 5128 neonates born 5 years before the survey from the Ethiopian Demographic and Health Survey 2016 were reviewed. A multivariable logistic regression model was employed to assess the effect of women's autonomy and identify the determinate predictors of neonate death risk.

**Results:**

The rate of neonatal mortality in Ethiopia was 20.7 per 1000 live births). Women's hadn't autonomy in health care increase neonatal death by 2.72 times compared with those that had autonomy. Hadn't postnatal care was caused grown neonatal death by 5.48 times (AOR 5.48, 95% CI 1.29, 23.26). Delivering at a health institution had 0.61 times lowered neonatal death risk compared with delivering at of health institution without a health facility (AOR 0.61, 95% CI 0.38,0.97). Breastfeeding immediately within 1 h after birth had 0.17 times reduce neonatal death risk compared with not initiation of breastfeeding (AOR 0.17, 95% CI 0.12, 0.26). Women's gave birth single had 0.09 times reduced neonatal death risk than those that gave birth multiple (AOR 0.09, 95% CI 0.05, 0.18). Unknowingly, male neonates had a 1.84 times higher risk of death than females (AOR 1.84, 95% CI 1.20, 2.81).

**Conclusions:**

Neonatal mortality rate was significantly related to women's hadn't decided power on health care, hadn't postnatal care, delivered out of health institution, breastfed not immediately, and gave birth multiple. It is important to encourage mothers autonomy, use postnatal care service, and deliver in health institutions.

## Introduction

The first 28 days from birth is the most critical time of human life for diseases [1]. Globally yearly, 2.6 m infants die before reaching 1 month old. Moreover, one million of them will die on their first day and around 2.6 m are born dead. Mortality of children 1 month–5 years old have reduced significantly in the last decades. But in neonates, it was not significant with 7000 newborns dying daily. The reason is partially due to neonatal diseases are difficult to treat with one drug or intervention; neonates need a systematic approach [2]. Neonatal disease patterns and outcomes are important indicators for sufficient health care planning and the outcomes are the changes in health status on which nursing and medical care have direct influence.

Besides those programs, improving women's autonomy might be produce good outcome for neonate health. When we say women’s autonomy is the ability to access information and make decisions about one's own business [3–5]. Hence, women’s autonomy undoubtedly contributes to several health advantages for both the mother and their children [6] and it's a priority of proceeding generations. Evidence suggests that women in developing countries often have limited autonomy and control over their health regarded decisions [4]. Even though, both the maternal and neonatal health provision needs a multi-sector approach a robust decision-making autonomy of the mother is vital for reversing back the barriers at the household level [7]. Because, limited women’s decision-making power delays maternal healthcare utilization like antenatal care (ANC), postnatal care (PNC), and delivery health institutions [8–11]. Besides, limited participation in access to health services for their interest, lower involvement of women in a decision has also impact on the socio-economic, emotional, fertility decision, contraceptive use, and sexual lifetime of the women [12], which results in an entire population problem.

Studies show that the decision-making autonomy of females is low, specifically in developing countries. However, scaling up the women’s role in a decision leads to better uptake of healthcare access, poverty reduction, and household economic growth [4, 13–18]. Indeed, in low-income countries, over women's important role in the family, they essentially have seen as an ordinary homemaker.

In Ethiopia, studies show that maternal and neonatal health coverage is low [19]. According to the 2016 Ethiopian demographic health survey (EDHS 2016) report, only 11–18% of women were involved in making decisions alone, and 66–68% together with their husband or partner [20]. Also, the neonatal mortality rate in Ethiopia was 30death per 1000 live births [21]. Most importantly the first 28 days of life is the most vulnerable time for a child’s survival [22]. According to UNICEF, 2020 report, the death proportion of neonates globally on average is 17 death per 1000 live births in 2019, and it dropped by 52% from 38 deaths per 1000 in 1990. Furthermore, the report also shows that around 2.4 million children died in the first month of life, and closely 6700 children died every day of these about 33.33% of all neonatal deaths occurring within the first day after birth, and close to 75% occurring within the first week of birth in 2019 [22].

Despite a downing in the death rate of below the age of the first 28 days after birth in the globe, marked differences in death rates were found to vary from place to place in the world [22]. According to the recent UNICEF report, East Africa and South Asia, placed at the top in neonatal death with a projected rate of twenty-seven and twenty-five deaths per 1,000 live births in 2019, respectively [22]. The report also showed that sub-Saharan Africa neonates were ten times greater risk of death than among who was born in a developed world and the death risk of the neonate in the highest death country was 55 times higher than its counterpart. Of this event, more than 50% of neonate deaths happened in Ethiopia [22].

According to the sustainable development goal agenda of 2030 by improving maternal health care and engagement of women can achieve the reduction of maternal and neonate, infant, and child mortality. To achieve this agenda women's independence in decision-making plays a great role [23, 24]. However, In Ethiopia even though women's autonomy had this much importance their involvement in a decision is still left behind and there is no evidence of its impact on neonate death. Therefore, this study aimed to identify factors associated with neonatal death in Ethiopia. So as to provide a baseline data for further better studies that could be done in the near future especially in Ethiopia, to design appropriate management protocol and recommendations.

## Method

### Study area and setting

Ethiopia has a total population of 109.5 million in 2018. The 2007 Population and Housing Census results show that the population of Ethiopia grew at an average annual rate of 2.6% between 1994 and 2007, down from 2.8% during the period 1983–1994. Currently, the population growth rate is among the top ten countries in the world and is estimated to grow to over 210 million by 2060. According to UN estimations, life expectancy had improved substantially in recent years with male life expectancy reported to be 56 years and for women 60 years. The estimated number of infant mortality rates are relatively high, as 41 infants die (per 1000 live births). In Ethiopia, the neonate mortality rate is 30 death per (1000 live births) [21].

### Data source, sampling, and data collection

A population-based cross-sectional study was conducted at a national level in Ethiopia in 2016. The EDHS 2016 is the fourth and most recent in the Demographic and Health Survey series in Ethiopia [20]. This survey was conducted in nine regional states and two city administrations subdivided into 68 zones, 817 districts, and 16,253 kebeles (lowest local administrative units of the country).

The target group for this study was all neonates in Ethiopia, and those neonates in the selected enumeration areas (EA) were the study population and neonates from unselected enumeration areas were excluded from the study. Stratified two-stage cluster sampling was performed. Samples of EAs were selected independently in each stratum in two stages. At the primary stage, a total of, 260 EAs (82 in urban and 178 in rural areas) were selected with probability sampling proportional to EA size, and secondly, a fixed number of 30 and 36 households per cluster from urban and rural areas respectively were selected randomly. A total of 18,008 households were randomly selected, and 15,683 eligible women were interviewed. Complete information on 5128 live births was extracted from 2016 EDHS. The survey incorporated both women who were permanent household residents (a person's legal resident status in a country of which such person is not a citizen but where he or she has the right to reside permanently) as well as visitors of the household. The survey contained a variety of standard questionnaires recording information on basic socio-demographic information and detailed bio-medical information and women's autonomy.

### Outcome variable

The investigator used NMR as the outcome variable using the recommended definition as children age 28 days after birth per 1000 live births during a given year [22].

#### Independent variables

The independent variables included in this study were demographic and socioeconomic variables such as (age, religion, region, husband education, wealth status of households, place of residence, sources of drinking water, and availability of toilet facilities), maternal health characteristics like(age at first birth, number of children born, previous birth interval, sex of the child, type of birth, place of delivery, mode of delivery, PNC visits), nutritional assessments like when the child put to breastfed and Women's autonomy like( on access to education, own health care service, large household purchases, visits to family or relatives).

- The women’s autonomy measured based on the response to “Who makes the following decisions in the household about
- Obtaining own health care service?
- Large household purchases?
- Visits to family or relatives?

Response choices were: (a) women alone; (b) women and husband/partner; (c) women and other people; (d) husband/partner alone; (e) someone else; (f) other. This response groped into two as the value of 1 is assigned if the response was (a), (b), or (c), that is, the involvement of the respondent, or else 0, for no involvement of the respondent (d, e and f).

### Statistical analysis

For all analyses in this study sampling weight was employed to manage sampling error and non-responses observation. Further details on sample weights can be found in the EDHS report [22]. Descriptive statistics were employed to show the distribution of background characteristics. Binary logistic regression was done to see the association between each independent variable and outcome variable. A log-likelihood ratio (LR) was tested for the goodness of fit. All variables with *p* < 0.25 in the bivariate analysis were included in the final model of multivariable analysis to control all possible confounders. Variance inflation factor (VIF) > 10 and Tolerance (T) < 0.1 were considered as suggestive of the existence of multi co-linearity. A crude and adjusted odds ratio with 95%CI was estimated to identify determinates for the neonate death cases. In this study *p*-value < 0.05 was considered to declare a result as a statistically significant association. All analyses were performed using statistical software SPSS (version 25).

### Ethics approval

This study is a secondary analysis of a publicly available dataset where permission was obtained through registering with the DHS website and therefore no ethics approval was required.

## Results

### Socio-demographic characteristics of study participants

A total of 5128 women were included in this study. The median age of the participants was 31 years. The study participants (48.8%) were Muslim by religion and (69.2%) women had no education i.e. more than four out of six mothers. Similarly,(84.1%) women were rural residents, and (52.2%) husbands hadn't education. About (53.3%) of the participants were poor by the combined wealth index of the family. Moreover, around (69.5%) and (55.1%) of the participant were had unimproved drinking water sources and toilet facility respectively (Table 1).

**Table 1:** Socio-demographic, maternal health, nutritional and women's autonomy related characteristics of the study participants

Characteristics	%	N
*Women's autonomy*		
A decision on own health care		
Involvement of respondents	82.1	4212
Without the involvement of respondents	17.9	916
A decision on making large household purchases		
Involvement of respondents	75.7	3882
Without the involvement of respondents	24.3	1246
A decision on visits to family or relatives		
Involvement of respondents	80.3	4116
Without the involvement of respondents	19.7	1012
*Demographic and socioeconomic characteristics*		
Age group in years		
< 25	15.1	775
25–29	30.5	1562
30 or older	54.4	2791
Religion		
Orthodox	30.1	1541
Muslim	48.8	2504
Others	21.1	1083
Residence		
Urban	15.9	816
Rural	84.1	4312
Respondents Highest level of education		
No education	69.2	3550
Primary	23.4	1200
Secondary	5.1	259
Higher	2.3	119
Highest level of husbands/partner education		
No education	52.2	2676
Primary	32.6	1671
Secondary	8.8	449
Higher	6.5	332
Wealth index of the family		
Poor	53.3	2734
Medium	15.1	775
Rich	31.6	1619
Availability of toilet facility		
No	44.9	2304
Yes	55.1	2824
Source of drinking water		
Unimproved	69.5	3564
Improved	30.5	1564
*Reproductive history and neonate healthcare service-related characteristics*		
Place of delivery		
Respondents home	66.2	3396
Health facility	33.8	1732
Preceding birth interval in years		
< 2	20.6	1057
2–3	33.3	1710
4or more	46.0	2361
Age at first birth		
< 18	43.3	2218
18–25	55.4	2842
26+	1.3	68
Sex of child		
Male	51.3	2631
Female	48.7	2497
Plurality		
Single	98.3	5042
Multiple	1.7	86
Breastfeeding status		
Immediate	71.0	3640
Not immediate	29.0	1488
PNC check-up within two months		
No	91.7	4702
Yes	8.3	426
Neonate status within the 1st 28 days		
Alive	97.9	5022
Died	2.1	106

### Maternal health and nutritional related characteristics

From the total study participants, (98.3%) women had a parity of one, and (55.4%) women gave first birth at the age of 18 to 25 years. Moreover, (91.7%) of women hadn't postnatal checkup within 2 months for their most recent delivery (Table 1).

### Women's autonomy related characteristics

About 82.1%, 75.7%, and 80.3% of women had involvement in decision making on their health care, large household purchases, and family or relatives visits respectively (Table 1).

### Neonatal mortality rate

Results of this study showed that the neonatal mortality rate in Ethiopia was 20.7 deaths per (1000 live births). The NMR varied significantly with different settings. For instance, the NMR was higher among illiterate women 15 than among literate women 7 death per (1000 live births). Similarly, NMR with women who were not involved in decision making for their health care was 21 than women who do not involve in decision 19 death per (1000 live births) (Table 2).

**Table 2:** Neonatal mortality rates (per 1000 live births) distributions of the factors in Ethiopia

Characteristics	NMR
*Women's autonomy*	
A decision on own health care	
Involvement of respondents	19
Without the involvement of respondents	21
A decision on making large household purchases	
Involvement of respondents	15
Without the involvement of respondents	21
A decision on visits to family or relatives	
Involvement of respondents	17
Without the involvement of respondents	21
*Demographic and socioeconomic characteristics*	
Respondents age group in years	
< 25	3
25–29	7
30 or older	12
Religion	
Orthodox	6
Muslim	13
Others	3
Residence	
Urban	2
Rural	19
Respondents highest level of education	
No education	15
Primary	5
Secondary	1
Higher	1
Highest level of husbands/partner education	
No education	12
Primary	7
Secondary	1
Higher	1
Wealth index of the family	
Poor	12
Medium	5
Rich	3
Availability of toilet facility	
No	11
Yes	10
Source of drinking water	
Unimproved	15
Improved	6
*Reproductive history and neonate healthcare service-related characteristics*	
Place of delivery	
Respondents home	13
Health facility	7
Preceding birth interval in years	
< 2	6
2–3	6
4or more	9
Age at first birth	
< 18	11
18–25	9
26+	1
Sex of child	
Male	14
Female	7
Plurality	
Single	18
Multiple	3
Breastfeeding status	
Immediate	6
Not immediate	14
PNC check-up within two months	
No	21
Yes	1

### Factors associated with neonate death

From the multivariable logistic regression analysis, women's involvement in their healthcare-related decision-making autonomy, Place of delivery, age at first birth, neonate sex, Plurality, breastfeeding, and PNC had an association with neonatal death. Those women who hadn't been involved in their healthcare-related decision-making autonomy were 2.72 (AOR = 2.72; 95% CI 1.41, 5.24) times higher risk of neonate death risk compared to those who had involvement in maternal and child, neonate health-related decision making autonomy. The death risk of the neonate for those women who delivered at health institutions was 0.61 (AOR = 0.61; 95% CI 0.38, 0.97) times lower than among those women who delivered at their home. Similarly, the death risk of neonate among those women who hadn't PNC checkup was 5.48 (AOR = 0.61; 95% CI 0.38, 0.97) times higher compared to among those women who had PNC. Likewise, those women who had initiation of immediate breastfed were 0.17 (AOR = 0.17; 95% CI 0.12, 0.26) times lower the death risk of neonate than its counterpart. Moreover, the likelihood of neonate death among those women who had single birth were 0.09 (AOR = 0.09; 95% CI 0.05, 0.18) times lower compared to those women who had multiple births. Unlikely, the death risk of male neonates were 1.84 (AOR = 1.84; 95% CI 1.20, 2.81) times higher than female neonates. The death risk for those women who gave birth of age at first birth 18–25 years were 0.32 (AOR = 0.32; 95% CI 0.10,0.99) times lower compared to among those women who gave birth of age at first birth 26 and more (Table 3).

**Table 3:** Bivariable and multivariable analysis of factors associated with neonate death in Ethiopia (EDHS, 2016)

Selected predictors	Model I		Model II	
	COR	CI	AOR	CI
*Women's autonomy*				
A decision on own health care				
Involvement of respondents	1.00		1.00	
Without the involvement of respondents(ref.)	1.91*	1.02, 3.59	2.72**	1.41, 5.24
*Demographic and socioeconomic characteristics*				
Residence				
Urban			0.52	0.23, 1.20
Rural (ref.)			1.00	
Respondents age group in years				
< 25			0.61	0.31, 1.19
25–29			1.00	0.63, 1.59
30 or older (ref.)			1.00	
Religion				
Orthodox			1.19	0.57, 2.45
Muslim			1.94	0.99, 3.77
Others (ref.)			1.000	
Highest level of husbands/partner education				
No education			1.52	0.49, 4.72
Primary			1.65	0.53, 5.15
Secondary			0.77	0.18, 3.25
Higher (ref.)			1.00	
Wealth index of the family				
Poor			0.83	0.44, 1.54
Medium			1.03	0.52, 2.04
Rich (ref.)			1.00	
Availability of toilet facility				
No			1.12	0.68, 1.84
Yes (ref.)			1.00	
Source of drinking water				
Unimproved			0.70	0.45, 1.11
Improved (ref.)			1.00	
*Reproductive history and neonate healthcare service-related characteristics*				
Place of delivery				
Health facility			0.61*	0.38, 0.97
Respondents home			1.00	
Preceding birth interval in years				
< 2			1.62	0.95, 2.74
2–3			1.08	0.66, 1.77
4or more (ref.)			1.00	
Age at first birth				
< 18			0.48	0.16, 1.49
18–25			0.32*	0.10, 0.99
26+ (ref.)			1.00	
Sex of child				
Male			1.84**	1.20, 2.81
Female (ref.)			1.00	
Plurality				
Single			0.09***	0.05, 0.18
Multiple (ref.)			1.00	
Breastfeeding status				
Immediate			0.17***	0.12, 0.26
Not immediate (ref.)			1.00	
PNC check-up within two months				
No			5.48*	1.29, 23.26
Yes (ref.)			1.00	
Constant	0.014***		0.05*	
− 2log likelihood	1026.77		861.51	
Cox and Snell R square	0.001		0.033	

***Significant at *p* < 0.001; **p < 0.01; *p < 0.05

## Discussion

Results show that the neonatal mortality rate in Ethiopia was 20.7 per 1000 live births. This figure is higher than the previous work. Women's independence in a decision on their health care service was a contributor factor for neonate death in Ethiopia. A possible reason could be that autonomous mothers are more capable of accessing available health facilities and that they can greatly change the traditional balances of power and autonomy in familial relationships, with profound effects on child care like reduce their reproductive behavior risks, prolong birth intervals, utilize prenatal care, and immunize their children in general and result in lower neonatal mortality. Besides, women can make decisions on delaying the age at first birth which plays a valuable strategy to promote and improve neonatal health and survival. Usually, adolescent mothers face financial and social problems that cause less provision of kid care. Indeed, the physiological immaturity of teenage mothers causes more neonatal death since they may have a small uterus or narrow bony pelvis and lack social experience in caring for newborns. As a result of empowering, women in decision-making power might be answered all this problem. These findings are consistent with previous studies that assessed the effect of Women's autonomy on maternal and child health service utilization [3, 4, 6–8, 10–12]. Evidence from the previous studies in India has also confirmed that a women’s control over household resources (ability to keep money aside) has a significant positive effect on both the demand for prenatal care and the probability of hospital delivery [13].

The neonate death proportion for delivered at home was higher than delivered at health institutions. The difference might be happening due to a lack of adequate maternal and neonatal care at home. As a result, the newborn baby might not take the immediate nutrition after birth and it is also difficult to fix complications immediately if there is a problem while the mother delivered at home. These findings are supported by the previous studies that focused on the impact of institutional delivery on maternal health care as a result proper medical attention and hygienic conditions during delivery reduce the occurrence of complications and infections which might be caused to death or serious illness for the mother, baby, or both [5, 8, 17, 18, 21–24]. Indeed, not only delivered at home had defects but also delivered with week health facilities like no access of skilled providers, poor infrastructure, and substandard quality of care causes the risk of intrapartum-related deaths [1, 2, 9, 15]. However, quality of delivery services and variations in newborn care practices weren't included in these analyses but could affect the risk of neonatal deaths.

The sex of the newborn is also significantly associated with neonatal mortality. Unknowingly in this study being male are more likely to die than among those who are female. Even though, there is no previous study supported the present study the problem might be due to bad cultural practices of the community.

Similarly, the probability of neonatal mortality rate become increase as the number of plurality increases. This effect is mainly associated with the lower birth weight of twins or triplets, which in turn is one of the most important factors affecting neonatal survival. The reason might be that it needs extra demand for food during the first day of the neonate. Since breastfeeding is one of the main sources of nutrition, multiple births might cause neonates' lower calorie intake, and thus lower survival chances [3, 21]. Likewise, breastfeeding is a fundamental concern of proceeding the newborn's life, and the result of this study shown that breastfeeding has a significant positive effect on neonatal mortality. The possible reason might be the deleterious effects of infections related to neonatal deaths can be prevented by breast milk especially practicing breast milk-fed within the first 1hour after birth. This practice not only minimizing the death risk but also helps to wealth and brain development [24].

The death risk of neonate among those women who hadn't PNC checkup was 5.48 times higher compared to those women who had PNC. This might be the prompt postnatal care (PNC) for both the mother and the child is important to treat any complications arising from the delivery, as well as to provide the mother with important information on how to safeguard herself and her kid. Evidence from the previous studies on safe motherhood programs recommends that all women receive a check of their health within 2 days after delivery had a positive effect on the wellbeing of both mothers and neonates [21]. Concerning this, a study in India and rural Sierra Leone indicated that postnatal care has a significant positive effect on neonatal mortality [8, 13, 16, 19, 20, 23, 24].

## Strengths and limitations of the study

This study was based on the recent EDHS (2016)with a nationally representative large sample size. Moreover, this study applied both separated and simultaneously multivariable logistic regression modeling to handle the influence of Women's decision making autonomy alone on neonatal mortality and its associated factors based on the EDHS data. Despite the above strengths, the study might have recall bias since the participants were asked about the events that happened 5 years or more preceding the survey. The study also shares the restrictions of cross-sectional studies.

## Conclusion

This study showed that the neonatal mortality rate was high and thus the situation is alarming the country and need to be addressed by maternal and child health programs. Neonatal mortality was highly associated with women's autonomy, postnatal care service, place of delivery, breastfeeding, age at first birth, sex of the neonate, and the plurality of birth. The major causes of neonatal mortality were hadn't postnatal care visit, women's limited power on healthcare decision making, and lack of early initiations of breastfeeding as identified by this study. Therefore, many of those cases could be prevented by improving early identification of obstetric complications and immediate interventions, strengthen postnatal care services both at the community as well as in the health care institutions, screening the conditions early during intrapartum and postnatal period to give immediate measures to avoid preventable causes of neonatal mortality. The health professionals are responsible to provide quality postnatal care services for both the mother and the neonate both at health care institutions and in the community. The community is also responsible to seek health information during the prenatal and postnatal period which is provided by health professionals and put into practice. The government is also responsible to provide broadened access to women delivery throughout the country. It is important to encourage mothers independence in decision making on health care service in the family as well as in the community and promoting women's to put breast their child immediately after birth. Also, intervention program needs to be designed for age at first birth to be matured enough. Other scholars should incorporate some of the variables that are not addressed in this study such as distance to the health institutions, Mode of delivery, and cultural aspects. It is also very important if the mixed study design is applied.

## Data Availability

The data set supporting the conclusions of this article is held by the authors and the Central Statistical Agency, CSA, Ethiopia, and the de-recognized data may be made available if a unique request is crafted from the CSA website (https://www.csa.gov.et).

## Abbreviations

AOR: adjusted odds ratio
CI: confidence interval
EA: enumeration area
EDHS: Ethiopian Demographic and Health Survey
HIV/AIDS: human immune virus
NMR: neonatal mortality rate
PNC: postnatal care
PSU: primary sampling unit
SDG: sustainable development goal
SPSS: statistical package for social science
STI: sexually transmitted infections
